# Supplementary material for: Structural basis for Mis18 complex assembly and its implications for centromere maintenance
Source: EMBO Rep. 2024 Jul 1;25(8):13. doi: 10.1038/s44319-024-00183-w (PMC11315898; doi:10.1038/s44319-024-00183-w)
Supplement: Supplementary file 1 — Table EV1 [file 44319_2024_183_MOESM1_ESM.docx]

**Table EV1. Crystallographic Data collection and refinement statistics**

|  | **SeMet-**  **Mis18α/β_C-term_** | **Mis18α/β_C-term_** | **Mis18α_Yippee_** |
| --- | --- | --- | --- |
| Space group | C222_1_ | C222_1_ | P2_1_2_1_2 |
| Unit cell parameters (Å) | a=77.73  b=101.91  c=88.51  α=β=γ=90° | a=77.591  b=101.815  c=88.563  α=β=γ=90° | a=110.725  b=114.864  c=116.279  α=β=γ=90° |
| Wavelength (Å) | 0.97917 | 0.97856 | 1.12713 |
| **Data collection statistics** |  |  |  |
| Resolution range (Å) | 50.00-2.75  (2.75-2.80) | 50.00-2.50  (2.50-2.54) | 50.00-3.00  (3.05-3.00) |
| Number of unique reflections | 9402 (469) | 12467 (600) | 30063 (1479) |
| Completeness (%) | 99.4 (98.3) | 99.0 (96.0) | 99.3 (99.7) |
| R_merge_ | 0.387 (5.327) | 0.078 (0.919) | 0.105 (0.804) |
| R_pim_ | 0.112 (1.456) | 0.040 (0.447) | 0.055 (0.410) |
| Redundancy | 13.4 (12.7) | 5.2 (4.7) | 4.4 (4.5) |
| Mean I/σ | 8.7 (1.5) | 9.2 (1.8) | 16.5 (1.4) |
| **Refinement statistics** |  |  |  |
| Resolution range (Å) |  | 44.29-2.50 | 49.99-3.00 |
| R_work_/R_free_ (%) |  | 24.77/27.96 | 20.26/25.00 |
| RMSD bonds (Å) |  | 0.008 | 0.012 |
| RMSD angles (deg) |  | 1.069 | 1.294 |
| Average B factor (Å^2^) |  | 91.18 | 83.84 |
| Number of water molecules |  | 17 | 8 |
| Ramachandran favoured (%) |  | 97.89 | 95.51 |
| allowed (%) |  | 2.11 | 4.49 |
| not allowed (%) |  | 0 | 0 |
