# Supplementary material for: Structural basis for Mis18 complex assembly and its implications for centromere maintenance
Source: EMBO Rep. 2024 Jul 1;25(8):13. doi: 10.1038/s44319-024-00183-w (PMC11315898; doi:10.1038/s44319-024-00183-w)
Supplement: Supplementary file 2 — Table EV2 [file 44319_2024_183_MOESM2_ESM.docx]

**Table EV2. Summary of SAXS data**

|  | **Mis18**  **α/β ΔN** | **Mis18α/β** | **Mis18_Core_** |
| --- | --- | --- | --- |
| **SASBDB accession** |  |  |  |
|  |  |  |  |
| **Guinier analysis** |  |  |  |
| *I(0)* (cm^-1^) | 0.026  ±7.1x10^-5^ | 0.057  ±1.0x10^-4^ | 0.25  ±6.9x10^-4^ |
| *Rg* (Å) | 53  ±0.21 | 60  ±0.17 | 63  ±0.24 |
| *Rc* (Å) | 26 | 30 | 31 |
| *q_min_* (Å^-1^) | 0.0060 | 0.0090 | 0.0093 |
|  |  |  |  |
| **P(r) analysis** |  |  |  |
| *I(0)* (cm^-1^) | 0.026  ±6.4x10^-5^ | 0.056  ±1.1x10^-4^ | 0.25  ±6.3x10^-4^ |
| *Rg* (Å) | 55  ±0.17 | 60  ±0.16 | 65  ±0.20 |
| *D_max_* (Å) | 190 | 215 | 230 |
| Porod volume *(*Å^3^) | 213302 | 377272 | 500371 |
| MW from Porod volume (kDa) | 125 | 222 | 294 |
| *V_C_ (*Å^2^) | 888 | 1161 | 1245 |
| MW from *V_C_* (kDa) | 120 | 192 | 220 |
|  |  |  |  |
| ***DAMMIN ab initio* modelling**  **(30 models)** |  |  |  |
| Symmetry | P1 | P1 | P1 |
| *NSD* mean and s.d. | 0.693  ±0.015 | 0.668  ±0.017 | 0.731  ±0.018 |
| *χ^2^* (reference model) | 1.27 | 1.33 | 0.997 |
|  |  |  |  |
| ***DAMMIN ab initio* modelling**  **(30 models)** |  |  |  |
| Symmetry | P2 | P2 | P2 |
| *NSD* mean and s.d. | 0.858  ±0.096 | 0.824  ±0.046 | 0.937  ±0.110 |
| *χ^2^* (reference model) | 1.26 | 1.33 | 0.976 |
