# Supplementary material for: Structural basis for Mis18 complex assembly and its implications for centromere maintenance
Source: EMBO Rep. 2024 Jul 1;25(8):13. doi: 10.1038/s44319-024-00183-w (PMC11315898; doi:10.1038/s44319-024-00183-w)
Supplement: Supplementary file 3 — Table EV3 [file 44319_2024_183_MOESM3_ESM.docx]

**Table EV3. Summary of Primers Used for Site Directed Mutagenesis**

|  | **Forward Primer** | **Reverse Primer** |
| --- | --- | --- |
| **Human Expression Vector** |  | |
| Mis18α_I201A/L205A_ | 5’-tgaaagcagagttgaagcagaaaagtctgcaacacagatggaagat-3’ | 5’-atcttccatctgtgttgcagacttttctgcttcaactctgctttca-3’ |
| Mis18α_I201D/L205D_ | 5’-gaaagcagagttgaagatgaaaagtctgatacacagatggaagat-3’ | 5’-atcttccatctgtgtatcagacttttcatcttcaactctgctttc-3’ |
| Mis18α_L212A/L215A/L219A_ | 5’-acagatggaagatgtcgcgaaagcagcacaaatgaaggcgtgggaggccg-3’ | 5’-cggcctcccacgccttcatttgtgctgctttcgcgacatcttccatctg-3’ |
| Mis18α_E103R/D104R/T105A_ | 5’-agctgggtggccagccagaggcgcgccaactgcatcctgcttc-3’ | 5’-gaagcaggatgcagttggcgcgcctctggctggccacccagct-3’ |
| Mis18β_L199D/I203D_ | 5’-aaaagattgcagaggataaagagaaggatgtgctaacgcacaatcgc-3’ | 5’-gcgattgtgcgttagcacatccttctctttatcctctgcaatcttt-3’ |
| **Bacterial Expression Vector** |  | |
| Mis18α_C154R/D160R_ | 5’-aatctgggttatgtttatcgtcgtaccccgaaaaacctg-3’  5’-cgtcgtaccccgaaaaacctgcgttataaacgtgacctgttttg-3’ | 5’-caggtttttcggggtacgacgataaacataacccagatt-3’  5’-caaaacaggtcacgtttataacgcaggtttttcggggtacgacg-3’ |
| Mis18α_I201A/L205A_ | 5'-aacctggaaagccgtgtggaagctg  aaaaaagcgcgacccagatggaagatg-3' | 5'-catcttccatctgggtcgcgctttttt  cagcttccacacggctttccaggtt-3' |
| Mis18α_L212A/L215A/L219A_ | 5'-cctgacccagatggaagatgttgcgaaagc  agcgcagatgaaagcgtgggaagcaga-3' | 5'-tctgcttcccacgctttcatctgcgct  gctttcgcaacatcttccatctgggtcagg-3' |
| Mis18β_L199D/I203D_ | 5'-tccgctgagcgaaaaaattgccgaagataaa  gaaaaagacgtgctgacccataatcgtct-3' | 5'-agacgattatgggtcagcacgtcttttt  ctttatcttcggcaattttttcgctcagcgga-3' |
