# Supplementary figures and images for: Structural basis for Mis18 complex assembly and its implications for centromere maintenance
Source: EMBO Rep. 2024 Jul 1;25(8):13. doi: 10.1038/s44319-024-00183-w (PMC11315898; doi:10.1038/s44319-024-00183-w)

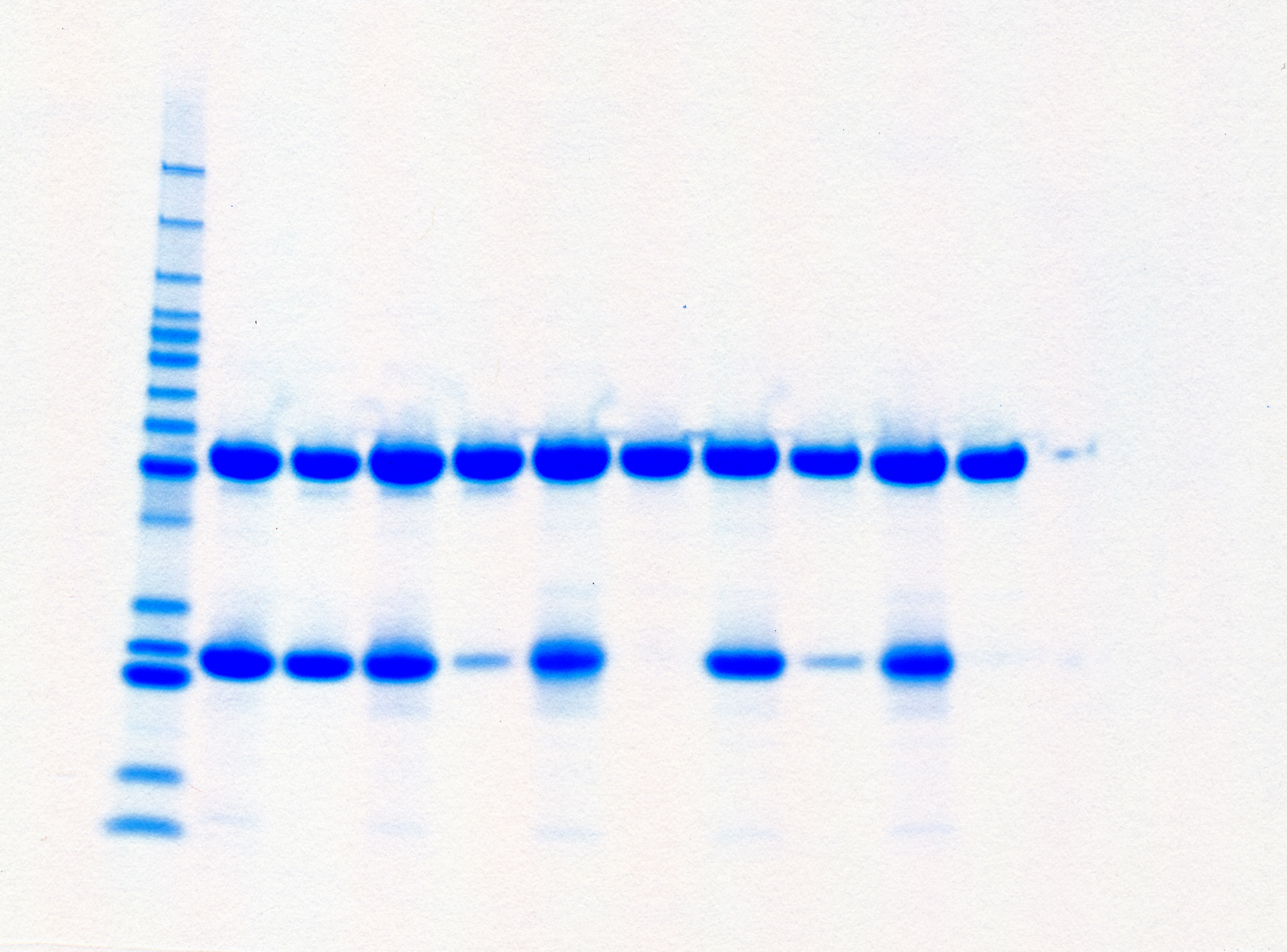

Supplement: Supplementary file 6 — Source data Fig. 4 [file 44319_2024_183_MOESM6_ESM.zip › Figure4/4A/Mis18 Interaction SDS PAGE.tif]

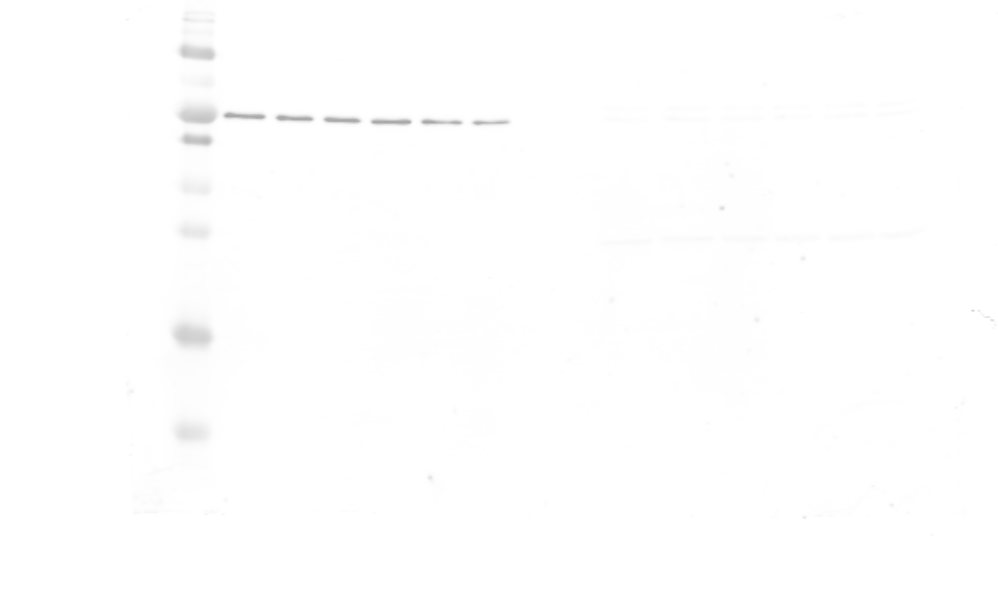

Supplement: Supplementary file 6 — Source data Fig. 4 [file 44319_2024_183_MOESM6_ESM.zip › Figure4/4A/Inverted LICOR Western tubulin.tif]

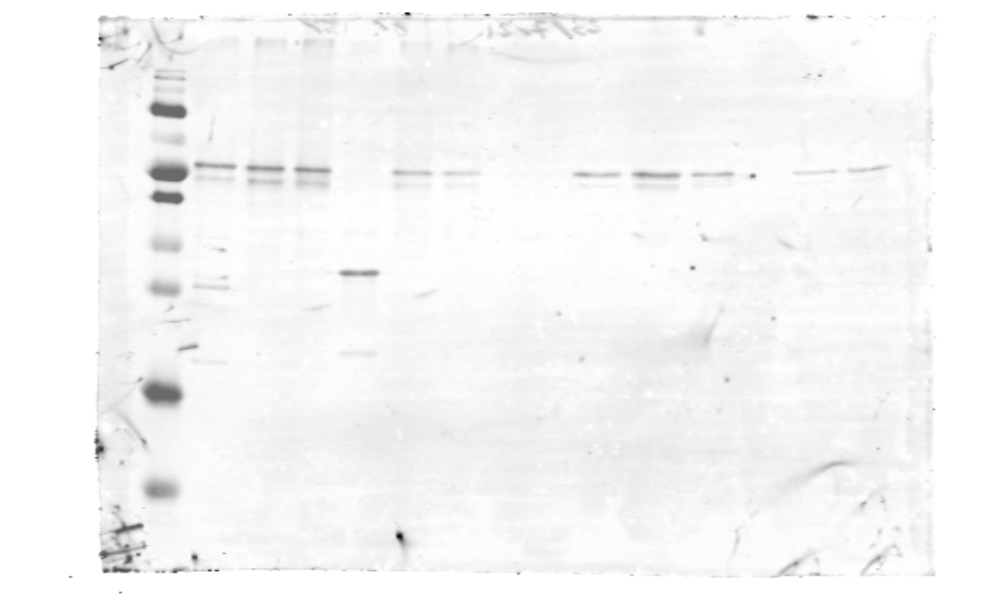

Supplement: Supplementary file 6 — Source data Fig. 4 [file 44319_2024_183_MOESM6_ESM.zip › Figure4/4A/Inverted LICOR Western mCherry.tif]

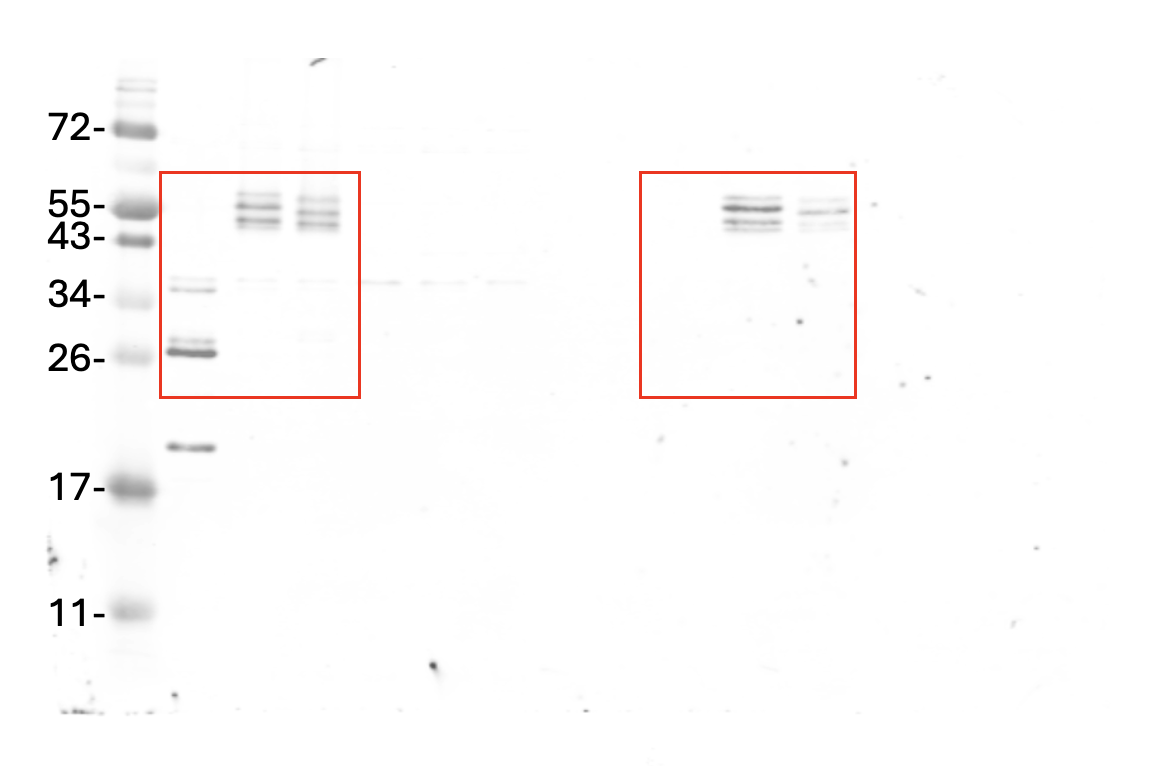

Supplement: Supplementary file 6 — Source data Fig. 4 [file 44319_2024_183_MOESM6_ESM.zip › Figure4/4A/Labeled LICOR Western GFP.png]

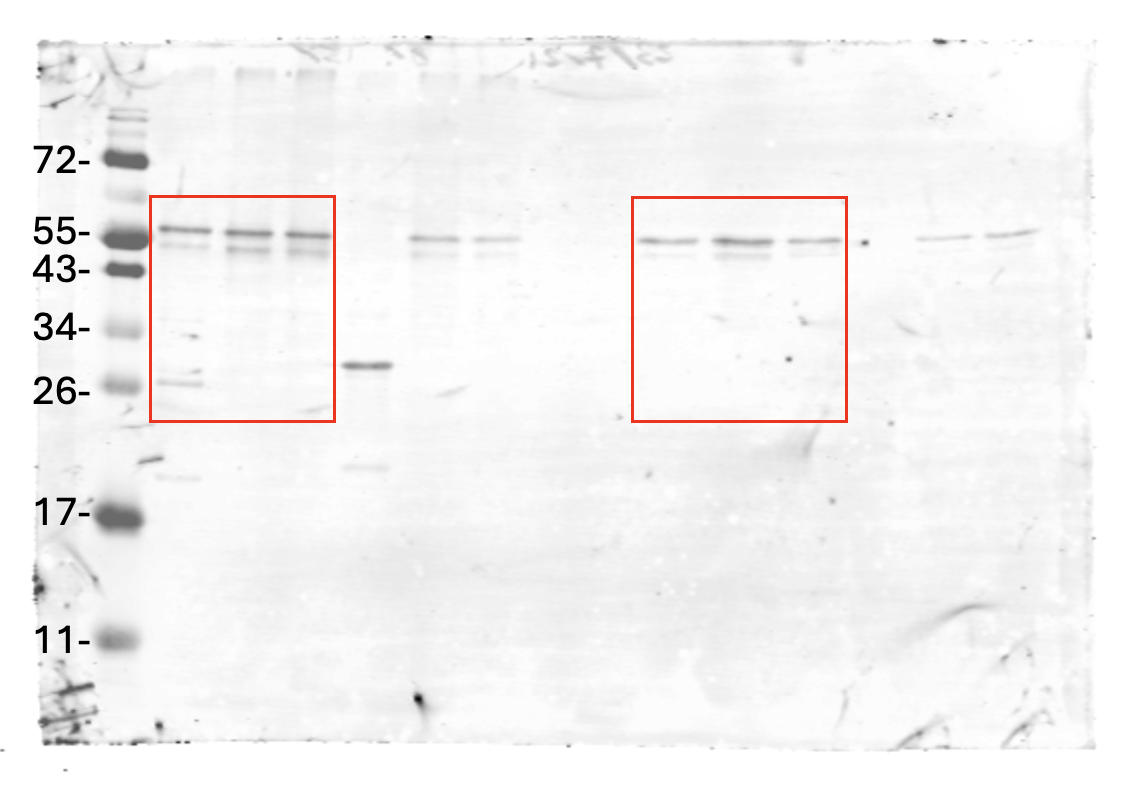

Supplement: Supplementary file 6 — Source data Fig. 4 [file 44319_2024_183_MOESM6_ESM.zip › Figure4/4A/Labeled LICOR Western mCherry.png]

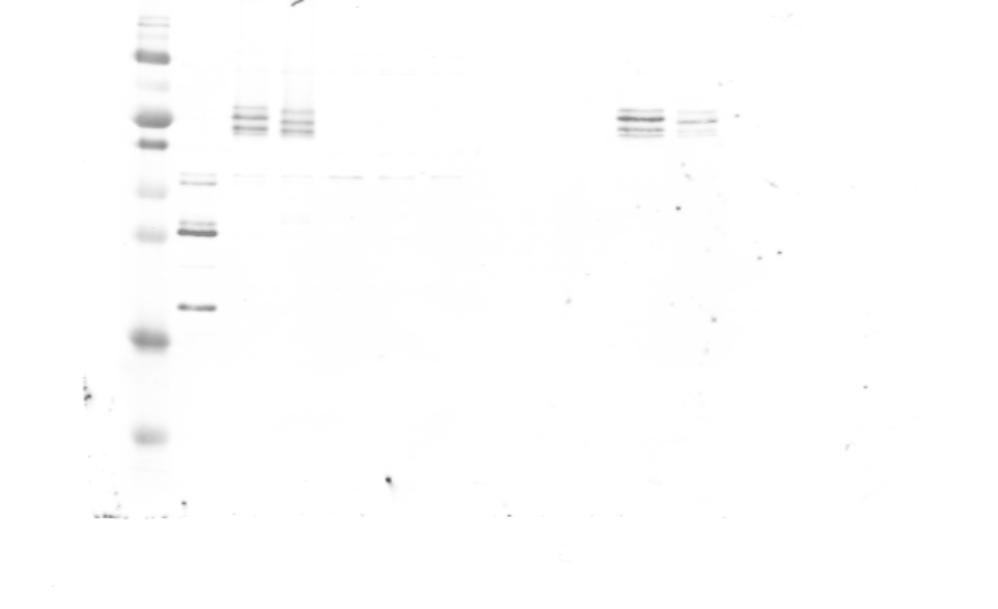

Supplement: Supplementary file 6 — Source data Fig. 4 [file 44319_2024_183_MOESM6_ESM.zip › Figure4/4A/Inverted LICOR Western GFP.tif]

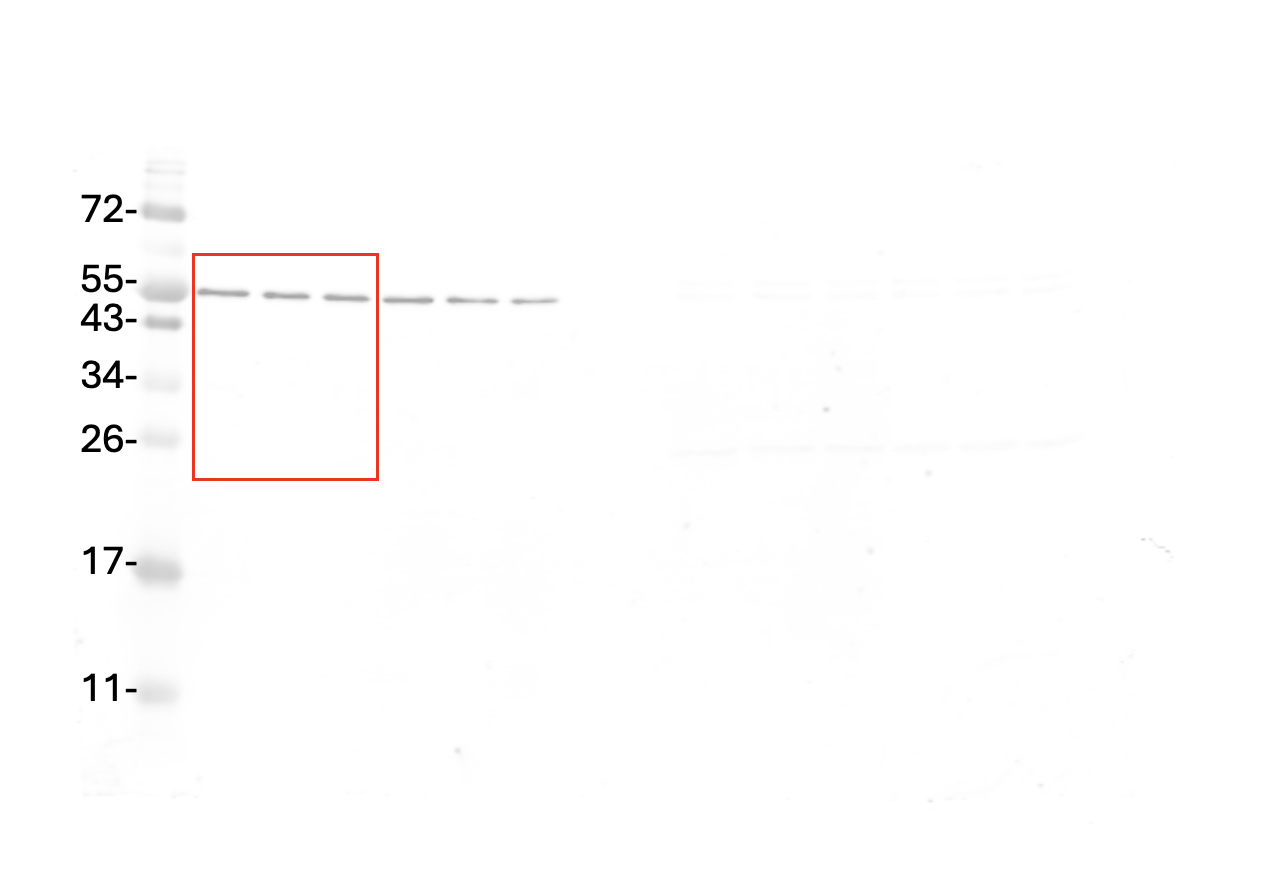

Supplement: Supplementary file 6 — Source data Fig. 4 [file 44319_2024_183_MOESM6_ESM.zip › Figure4/4A/Labeled LICOR Western tubulin.png]
