# Supplementary material for: Structural basis for Mis18 complex assembly and its implications for centromere maintenance
Source: EMBO Rep. 2024 Jul 1;25(8):13. doi: 10.1038/s44319-024-00183-w (PMC11315898; doi:10.1038/s44319-024-00183-w)
Supplement: Supplementary file 8 — Expanded View Figures [file 44319_2024_183_MOESM8_ESM.pdf]

Expanded View Figures

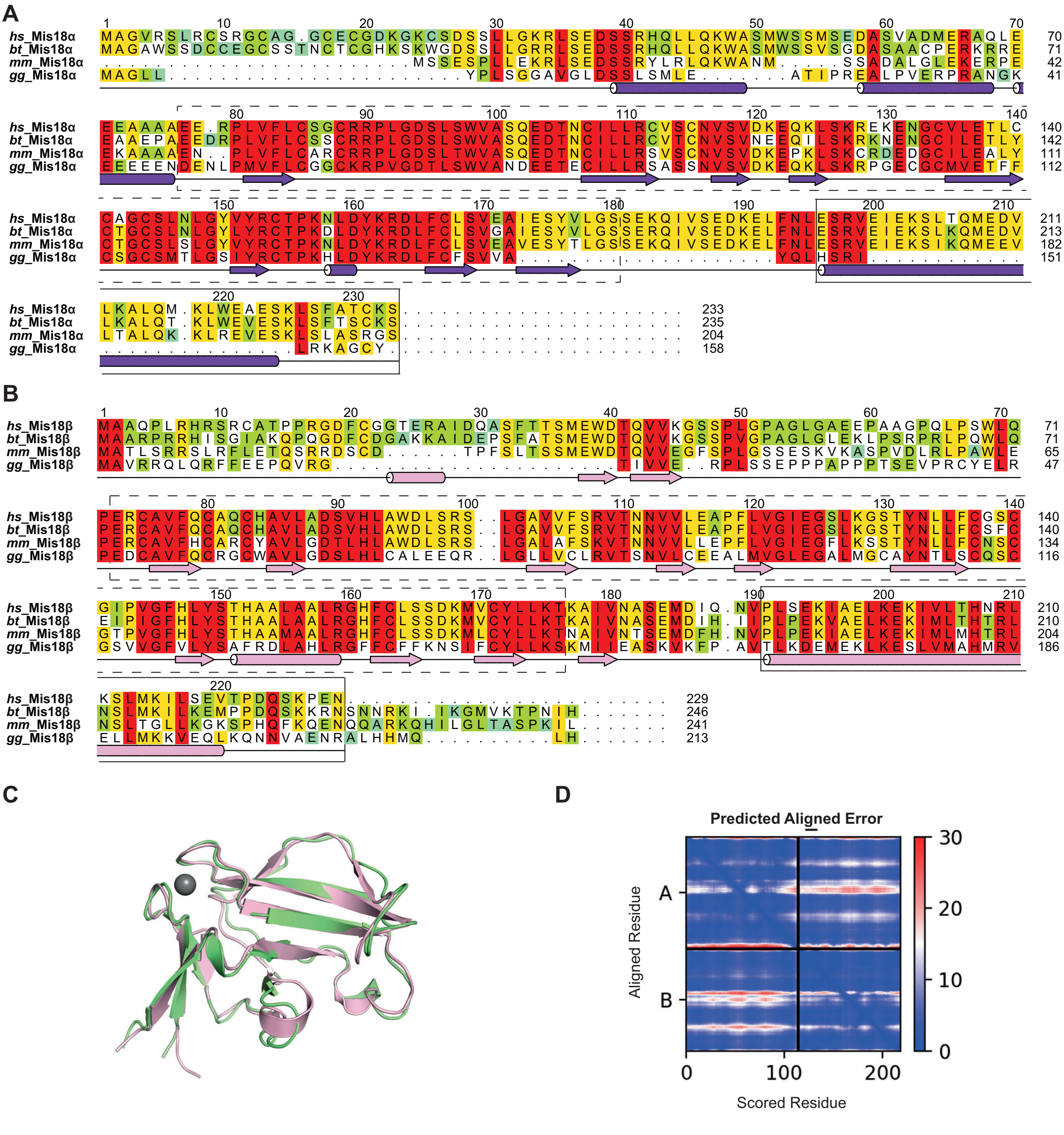

Figure EV1. Mis18α and Mis18β contain two domains capable of oligomerising.

(**A**, **B**) Domain architecture and amino acid conservation of (**A**) Mis18α and (**B**) Mis18β. Alignments include *Homo sapiens* (*hs*), *Bos taurus* (*bt*), *Mus musculus* (*mm*) and *Gallus gallus* (*gg*). The conservation score is mapped from red to cyan, where red corresponds to highly conserved and cyan to poorly conserved. Secondary structures as annotated/predicted by Conserved Domain Database [CDD] and PsiPred, http://bioinf.cs.ucl.ac.uk/psipred. Multiple sequence alignments were performed with MUSCLE (Madeira et al, 2019) and edited with Aline (Bond and Schüttelkopf, 2009). Dashed boxes highlight Yippee domains whilst solid boxes highlight C-terminus α-helices. (**C**) Superposition of $Mis18β_{Yippee}$ structures predicted by AlphaFold (light pink) and RaptorX (green). RaptorX generated five models and the model with the lowest estimated error (1.9 Å) is shown here. The AlphaFold and RaptorX models superpose well with an RMSD of 0.95 Å. (**D**) The PAE plot corresponding to the $Mis18α/β_{Yippee}$ AlphaFold model shown in Fig. 1D.

 © The Author(s)

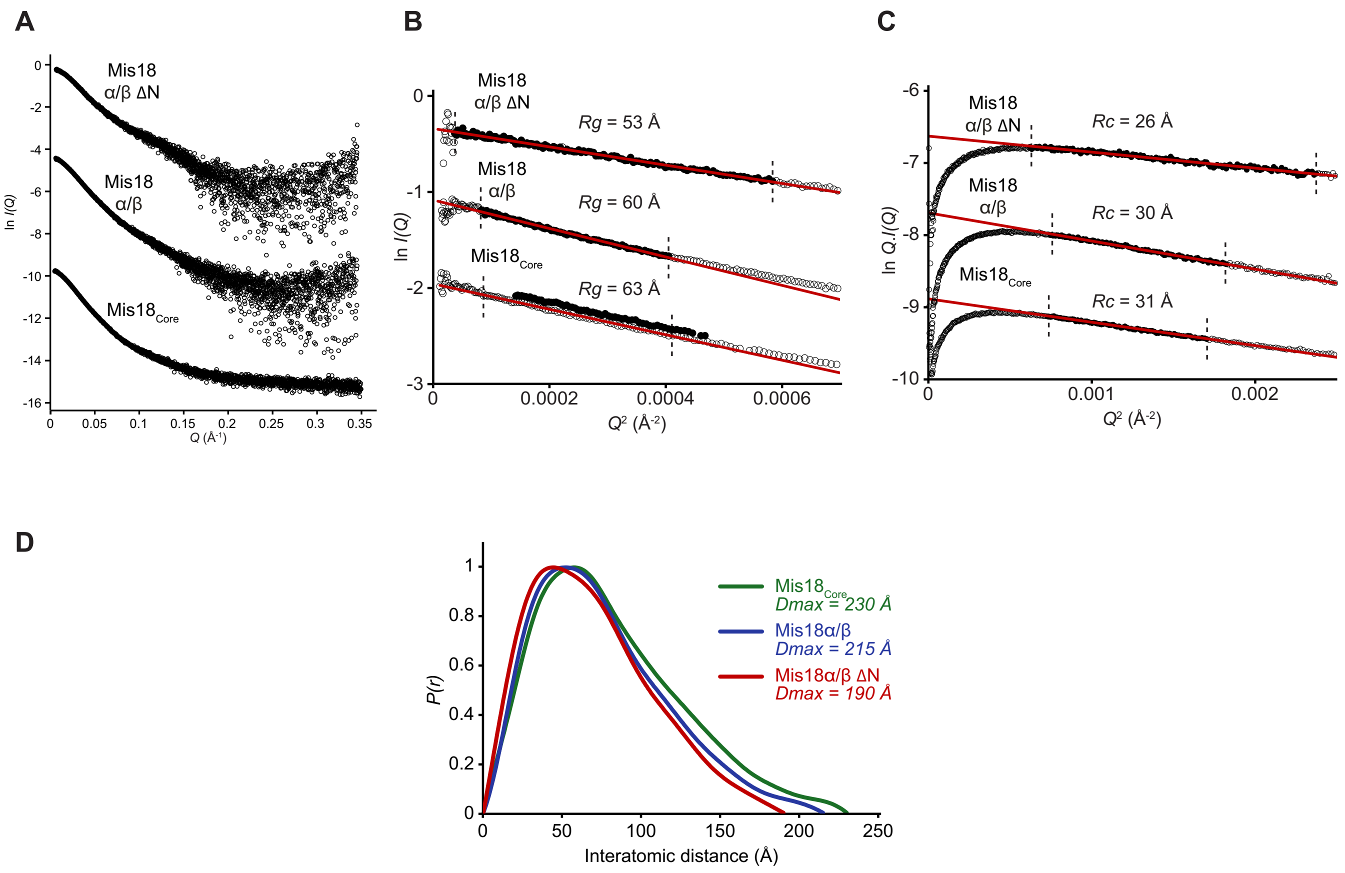

Figure EV2. SAXS analysis of Mis18α/β ΔN, Mis18α/β and $Mis18_{core}$.

(**A**) SAXS scattering curves of Mis18α/β ΔN, Mis18α/β and $Mis18_{core}$. (**B**) Guinier Plot showing *Rg* of 53 Å, 60 Å, and 63 Å for Mis18α/β ΔN, Mis18α/β and $Mis18_{core}$, respectively. (**C**) Modified Guinier Plot showing *Rc* of 26 Å, 30 Å, and 31 Å for Mis18α/β ΔN, Mis18α/β and $Mis18_{core}$, respectively. (**D**) SAXS *P(r)* distributions showing maximum dimensions of 190 Å, 215 Å, and 230 Å for Mis18α/β ΔN, Mis18α/β and $Mis18_{core}$, respectively.

© The Author(s)

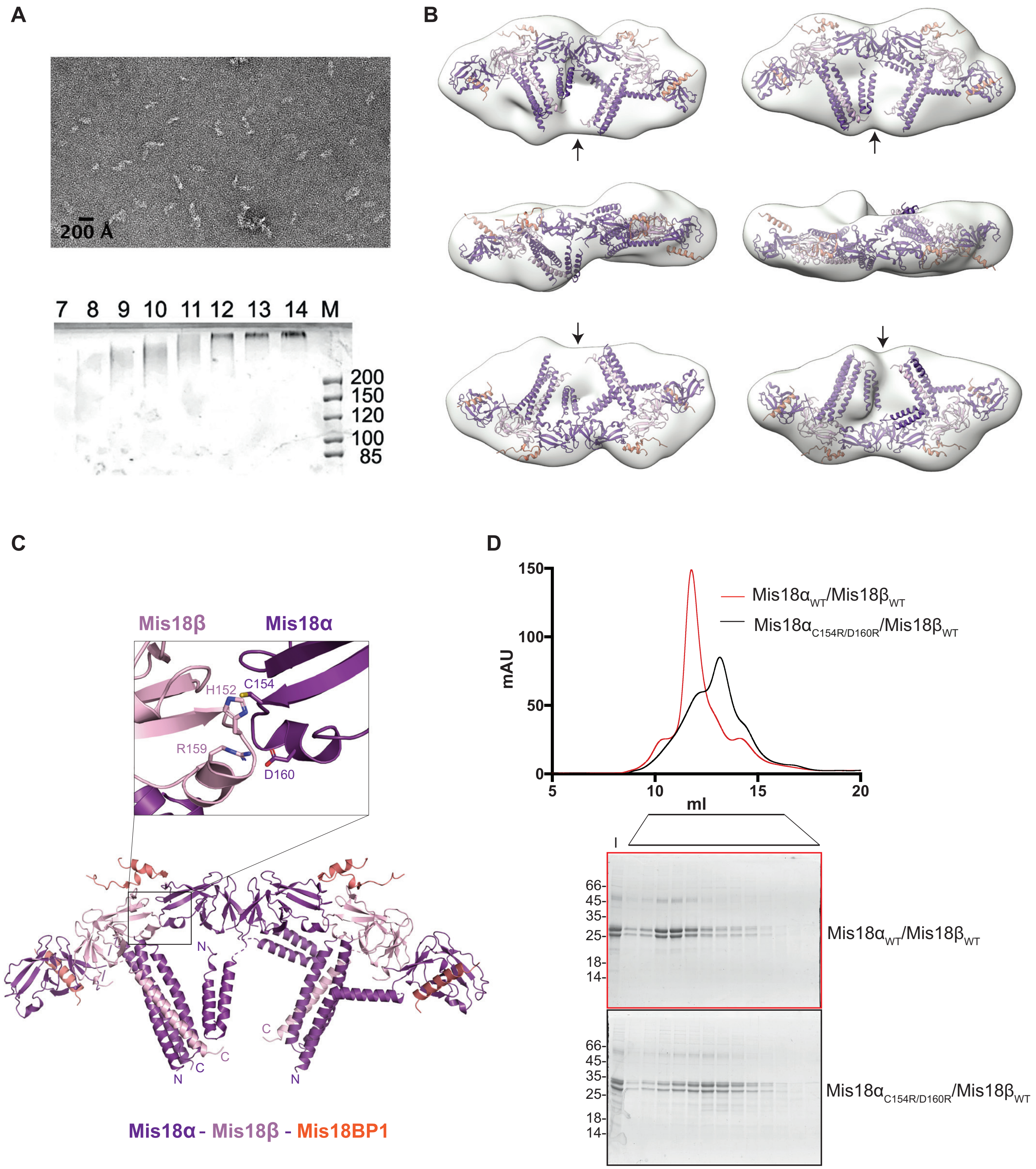
A
200 Å
7 8 9 10 11 12 13 14 M
200
150
120
100
85
B
C
Mis18β
Mis18α
H152
C154
R159
D160
N
C
Mis18α - Mis18β - Mis18BP1
D
150
100
50
0
mAU
5
10
15
20
ml
Mis18α$_{WT}$/Mis18β$_{WT}$
Mis18α$_{C154R/D160R}$/Mis18β$_{WT}$
I
66-
45-
35-
25-
18-
14-

 © The Author(s)

Figure EV3. Structural characterisation of the Mis18$_{core}$ complex.

(**A**) Representative micrograph of negative staining EM of the Mis18α/Mis18β/Mis18BP1$_{20\text{-}130}$ (Mis18$_{core}$) complex cross-linked using GraFix (Kastner et al, 2008; Stark, 2010). Beneath is the corresponding SDS-PAGE analysis of fractions from GraFix, fractions 8 and 9 were used to make grids. (**B**) Two models (Class II-III) generated for Mis18$_{core}$ from negative staining EM analysis. All show that the overall shapes of the Mis18$_{core}$ resemble a telephone handset with 'ear' and 'mouth' pieces assuming different relative orientations. (**C**) Cartoon representation of the model of Mis18$_{core}$ complex generated in Fig. 2B. Zoomed in panel shows interaction between Mis18α and Mis18β Yippee domains using the second interface. Important residues for this interaction highlighted in pink and purple. (**D**) SEC profile of Mis18α$_{WT}$/Mis18β$_{WT}$ (red) and Mis18α$_{C154R/D160R}$/Mis18β$_{WT}$ (black) and corresponding SDS-PAGE analysis of the fractions. Samples were analysed using Superdex 200 increase 10/300 in 20 mM Tris-HCl pH 8.0, 250 mM NaCl and 2 mM DTT.

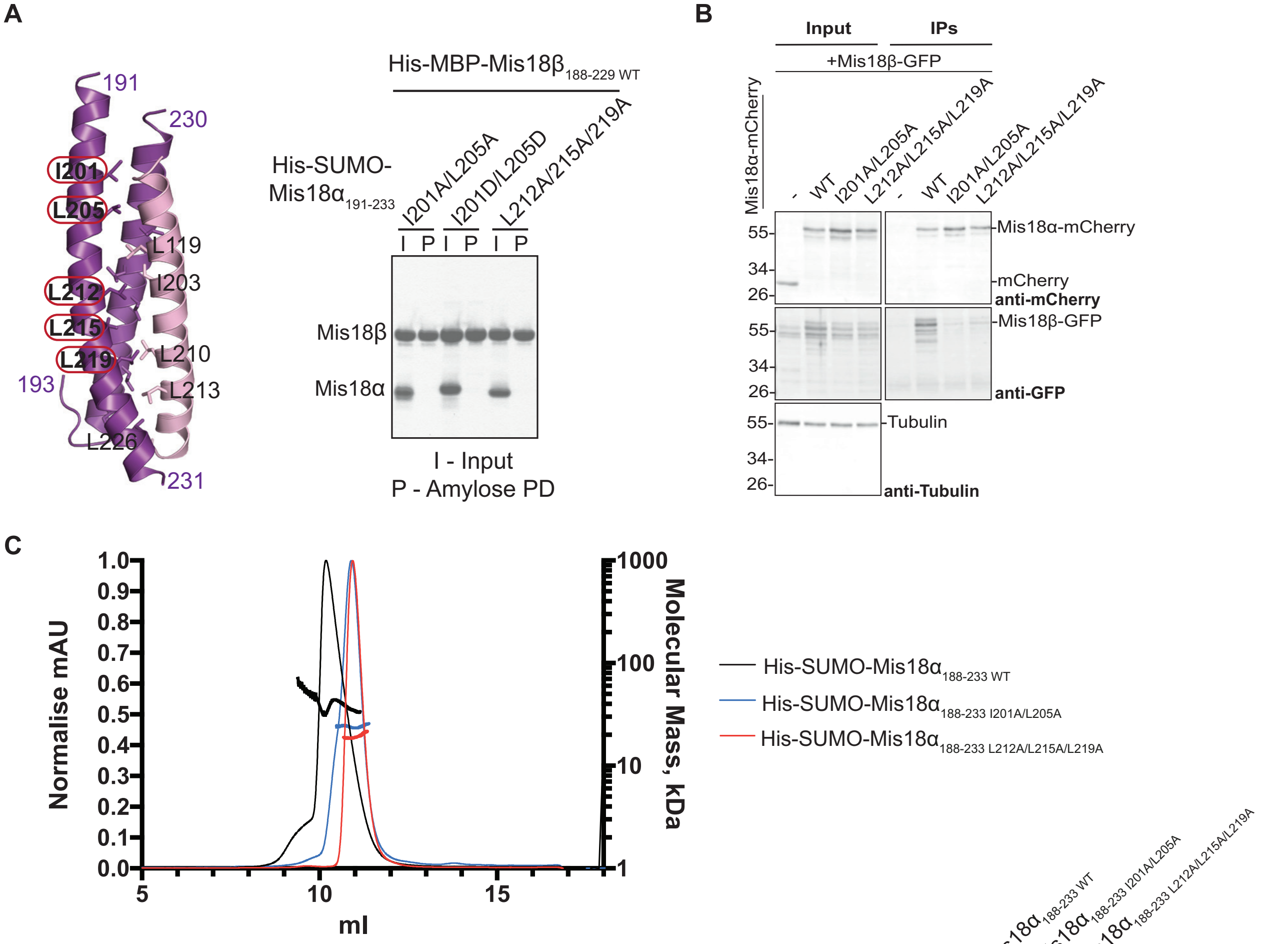

	Measure MW in kDa (Calculated MW)	Number of Subunits
His-SUMO-Mis18α$_{188\text{-}233\ WT}$	38.8±0.7 (19.5)	Dimer
His-SUMO-Mis18α$_{188\text{-}233\ I201A/L205A}$	24.1±0.4 (19.5)	Monomer
His-SUMO-Mis18α$_{188\text{-}233\ L212A/L215A/L219A}$	19.5±0.4 (19.4)	Monomer

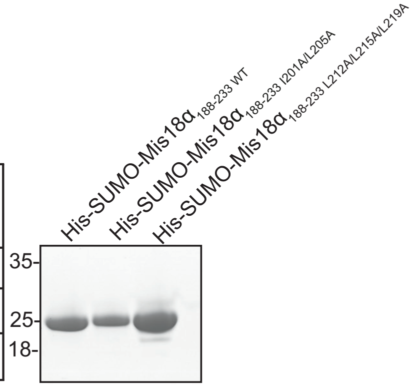

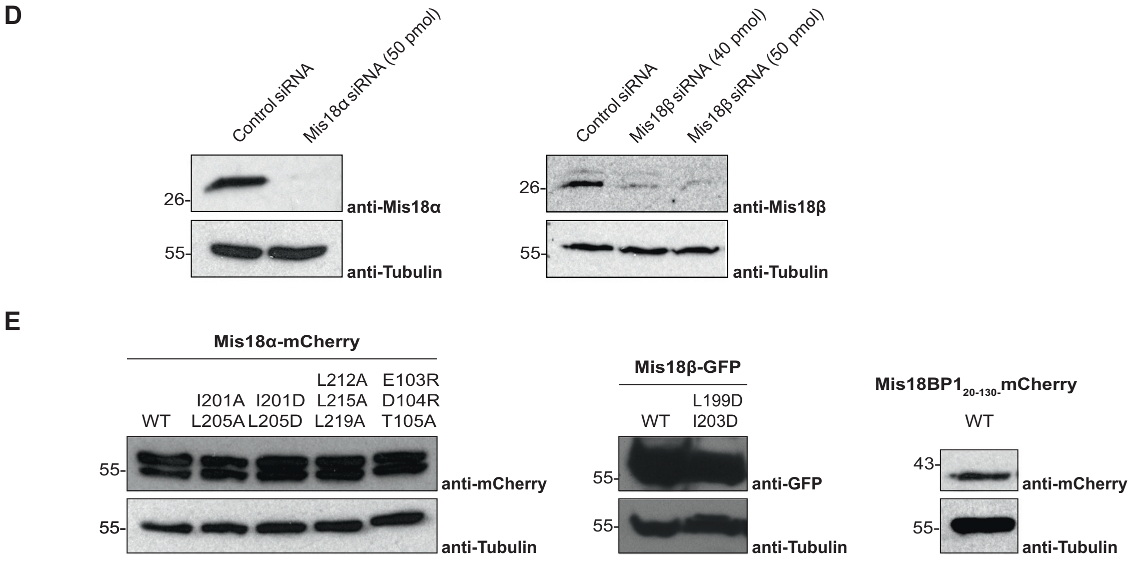

 © The Author(s)

Figure EV4. Structural and biochemical characterisation of Mis18α C-terminal helix.

(**A**) Cartoon representation of the crystal structure of Mis18$\alpha_{C\text{-}term}$/Mis18$\beta_{C\text{-}term}$ (PDB ID: 7SFY). Mis18α is shown in purple and Mis18β in light pink. Potential residues involved in the interaction are highlighted. Mis18α (purple) and Mis18β (light pink). Right panel shows SDS-PAGE analysis of cobalt and amylose pull-down of His-MBP-Mis18$\beta_{188\text{–}229\ WT}$ with His-SUMO-Mis18$\alpha_{191\text{–}233}$ mutants. SDS-PAGE shows protein bound to nickel resin as input (I) and protein-bound to amylose resin to assess interaction (P). Control with WT proteins shown in Fig. 4A. (**B**) Western blot analysis of co-immunoprecipitation (Co-IP) experiments using Mis18α antibody to test interaction of mCherry as a control, Mis18α−mCherry with and without mutations in the C-terminal α-helices and Mis18β-GFP. Top panel shows blot against mCherry, middle panel shows blot against GFP, and bottom panel shows blot against tubulin as loading control. (**C**) SEC-MALS of His-SUMO-Mis18$\alpha_{188\text{-}233\ WT}$, His-SUMO-Mis18$\alpha_{188\text{-}233\ I201A/L205A}$ and His-SUMO-Mis18$\alpha_{188\text{-}233\ L212A/L215A/L219A}$. Normalised absorption at 280 nm (mAU, left y-axis) and molecular mass (kDa, right y-axis) are plotted against elution volume (ml, x-axis). Measured molecular weight (MW) and the calculated subunit stoichiometry based on the predicted MW. Samples were analysed using a Superdex 75 increase in 50 mM HEPES pH 8.0, 150 mM NaCl and 1 mM TCEP. (**D**) Representative immunoblots showing expression levels of endogenous proteins after treatment with siRNA. (**E**) Representative immunoblots showing expression levels of transiently expressed tagged proteins after transfection.
